# A novel risk prediction nomogram for early death in patients with resected synchronous multiple primary colorectal cancer based on the SEER database

**DOI:** 10.1007/s00384-023-04435-4

**Published:** 2023-05-16

**Authors:** Xiangyu Zhang, Liang Zhao, Yanpeng Hu, Kai Deng, Wanbo Ren

**Affiliations:** Department of Gastrointestinal Surgery, Qilu Hospital of Shandong University Dezhou Hospital, 1751 Xinhu Street, Dezhou, 253000 China

**Keywords:** Synchronous multiple primary colorectal cancer, Early death, Nomogram, Surveillance, Epidemiology, And End Results (SEER)

## Abstract

**Background:**

Synchronous multiple primary colorectal cancer (SMPCC) involves the simultaneous occurrence of 2 or more independent primary malignant tumors in the colon or rectum. Although SMPCC is rare, it results in a higher incidence of postoperative complications and mortality compared to patients with single primary colorectal cancer (SPCRC).

**Methods:**

The clinical factors and survival outcomes of SMPCC patients registered on the Surveillance, Epidemiology, and End Results (SEER) database between 2000 and 2017 were extracted. The patients were divided into the training and validation cohorts using a ratio of 7:3. Univariate and multivariate logistic regression analyses were used to identify the independent risk factors for early death. The performance of the nomogram was evaluated using the concordance index (C-index), calibration curves, and the area under the curve (AUC) of a receiver operating characteristics curve (ROC). A decision curve analysis (DCA) was used to evaluate the clinical utility of the nomogram and standard TNM system.

**Results:**

A total of 4386 SMPCC patients were enrolled in the study and randomly assigned to the training (n = 3070) and validation (n = 1316) cohorts. The multivariate logistic analysis identified age, chemotherapy, radiotherapy, T stage, N stage, and M stage as independent risk factors for all-cause and cancer-specific early death. The marital status was associated with all-cause early death, and the tumor grade was associated with cancer-specific early death. In the training cohort, the nomogram achieved a C-index of 0.808 (95% CI, 0.784–0.832) and 0.843 (95% CI, 0.816–0.870) for all-cause and cancer-specific early death, respectively. Following validation, the C-index was 0.797 (95% CI, 0.758–0.837) for all-cause early death and 0.832 (95% CI, 0.789–0.875) for cancer-specific early death. The ROC and calibration curves indicated that the model had good stability and reliability. The DCA showed that the nomogram had a better clinical net value than the TNM staging system.

**Conclusion:**

Our nomogram can provide a simple and accurate tool for clinicians to predict the risk of early death in SMPCC patients undergoing surgery and could be used to optimize the treatment according to the patient's needs.

**Supplementary Information:**

The online version contains supplementary material available at 10.1007/s00384-023-04435-4.

## Background

Colorectal cancer (CRC) is the third most common malignancy and the second cause of cancer-related mortality worldwide [1]. Synchronous multiple primary colorectal cancer (SMPCC) is a rare subtype of CRC, characterized by the presence of two or more primary CRC lesions simultaneously or within 6 months from the detection of the first lesion [2]. SMPCC accounts for about 1.1% to 8.1% of all CRC cases [3]. Although SMPCC is rare, its incidence is increasing due to improvements in diagnostic imaging techniques such as digestive endoscopy and imaging techniques have led to a decline in the missed diagnosis rate of CRC lesions [4, 5]. Numerous studies have shown that the clinical features, pathological subtypes, pathogenesis, genetic mutations, and treatment outcomes tend to vary significantly between patients diagnosed with SMPCC and those diagnosed with single primary colorectal cancer (SPCRC) [6–8]. These findings suggest that treatment applied to SPCRC may be inappropriate to apply to patients diagnosed with SMPCC patients with SMPCC may benefit from a different treatment approach. Surgery remains the primary modality for the treatment of CRC. However, patients with SMPCC, especially those diagnosed with bilateral colon tumors and synchronous colon-rectum tumors, often require extensive surgical interventions, which may involve multiple colorectal segments, two or more anastomoses, and even total colectomy or proctectomy. As a result, patients with SMPCC tend to have a higher incidence of postoperative complications and mortality than those diagnosed with SPCRC [9]. However, although numerous studies have evaluated the long-term prognosis of SMPCC, relatively few studies have assessed the short-term outcome [10–12]. As a result, there is currently no effective tool that could be used to predict short-term mortality for SMPCC patients. Therefore in this study, we aimed to investigate the incidence of early death in surgically treated SMPCC patients based on data extracted from SEER database to identify the risk factors contributing to early death. Moreover, we developed and validated a nomogram to predict early death (survival time ≤ 3 months) to enable clinicians to optimize the treatment for SMPCC patients and hence reduce the incidence of early death.

## Methods

### Ethical considerations

The Surveillance, Epidemiology, and Results (SEER) program of the National Cancer Institute provides cancer incidence and survival data from 18 established cancer registries which cover approximately 30 percent of the population in the United States. Since SEER is a public domain database, patient informed consent and ethical clearance were not required to conduct this study. The research complied with all relevant ethical criteria and was conducted in line with the "Declaration of Helsinki" in 1964.

### Selection criteria

**Fig. 1:**
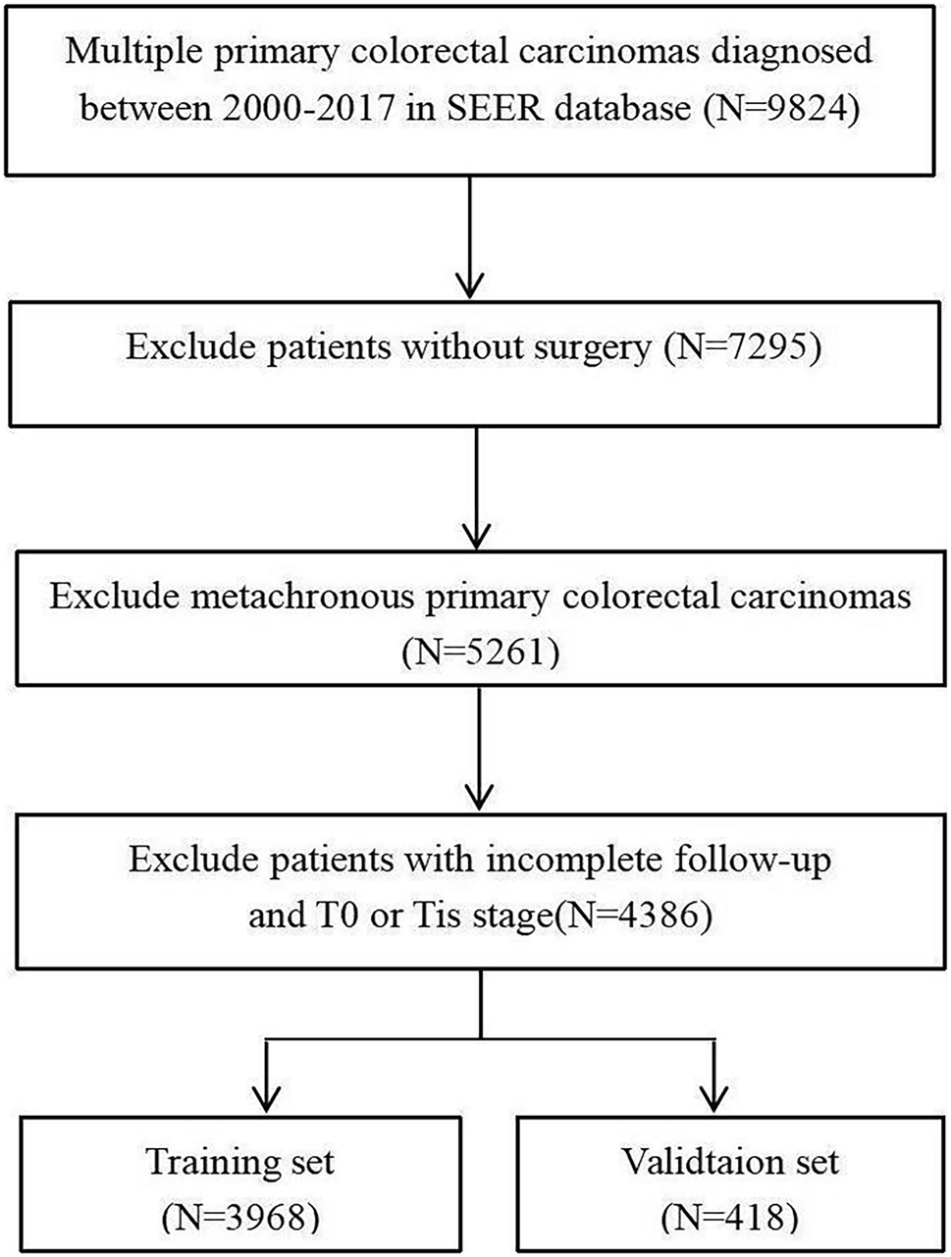
Flowchart illustrating the patient selection process

The SEER*Stat version 8.4.0.1 software was used to extract the demographic, clinical, and survival data of SMPCC patients registered on the SEER database between 2000 and 2017. The inclusion criteria were as follows: (1) Two or more primary CRC lesions diagnosed in the same patient; (2) Pathologically confirmed adenocarcinoma; (3) The diagnosis interval for the identification of the different primary CRC lesions of less than 6 months [13]. The exclusion criteria were; (1) Age less than 18 years old; (2) Previous history of other malignant tumors; (3) No surgical treatment; (4) Patients diagnosed only by autopsy or death certificate; (5) Diagnosed with a carcinoma-in-situ; (6) Cases with missing survival information and insufficient follow-up. In addition, the patients who did not undergo surgical interventions or had a CRC diagnosis following an autopsy were also excluded. The patient selection process is illustrated in Fig. 1.

### Data extraction

The CRC were coded as defined by the International Classification of Cancer Diseases (ICD-O-3). Codes C18.0 to C18.9 refer to colon tumors, C19.9 to rectosigmoid tumors and C20.9 to rectal tumors. All tumors were divided into 3 groups; right colon, left colon, and rectum. Tumors located between the cecum to the transverse colon were classified as right colon, while those located between the splenic flexure and the sigmoid colon were classified as left colon. Tumors encompassing the rectosigmoid junction and the rectum were classified as rectum. Patients were divided into 3 groups in accordance to the positional relationship of the multiple tumor lesions: unilateral group, bilateral group, and rectum-colon synchronous group. The unilateral group included patients with synchronous tumors located on one side (right-right colon, rectum-rectum, left-left colon), the bilateral group included patients with synchronous tumors on both sides (right-left colon, left–right colon), and the rectum-colon synchronous group included patients with synchronous tumors affecting the colon and the rectum (rectum-right colon; rectum-left colon). The lesion with the most advanced stage or size among the multiple lesions was used as the index tumor for analysis.

### Statistical analysis

All the patients were randomly assigned to the training and validation cohorts using a ratio of 7:3. The primary outcome measures for this study were early all-cause and cancer-specific early death within 3 months of diagnosis [14]. The categorical variables were expressed as numbers and percentages (n,%), and the differences in the distribution of the variables between the training and validation cohorts were assessed using Pearson's chi-square test. Univariate logistic regression analysis was performed on the training cohorts to identify the risk factors for all-cause and cancer-specific early death. The significant risk factors were included in the multivariate logistic regression analysis to identify the independent risk factors. The independent risk factors were then used to construct predictive nomograms for all-cause and cancer-specific early death. By mapping the value of each factor to the "points" axis, the points for early death probability for each variable were obtained. The total points can be calculated by summing them up [15].

The performance of the nomogram in the training and validation cohorts was evaluated as follows. The concordance index (C-index) was used to evaluate the nomogram's predictive performance, and a calibration curve with a 1000-times bootstrapping was plotted to evaluate the consistency between the actual and predicted probabilities. The area under the curve (AUC) with the 95% confidence interval (CI) of a receiver operating characteristic (ROC) curve was calculated to evaluate the discrimination ability of the nomogram. An area under the roc curve (AUC) value above 0.7 was considered to have good predictive capabilities [16]. Finally, a decision curve analysis (DCA) was performed to compare the clinical utility of the nomogram and standard AJCC TNM staging system. All statistical analyzes were carried out using the R software (version 4.1.2), and a two-sided p-value below 0.05 was deemed statistically significant.

## Results

### Patient characteristics

A total of 4386 SMPCC patients were enrolled in the study, of whom 53.92% (n = 2365) were males, and the rest were females (46.08%, n = 2021). Most patients were Caucasian (79.30%, n = 3478) and aged above 60 years (79.41%, n = 3483). The majority of the patients (66.53%, n = 2918) had bilateral tumors or rectum-colon synchronous tumors. Out of the 4386 SMPCC patients, 14.48% (n = 635) developed distant metastases, 34.72% (n = 1523) received chemotherapy, and 10.3% (n = 452) received radiotherapy. All-cause early death occurred in 9.07% (n = 398) of patients, while cancer-specific early death occurred in 6.00% (n = 263). The characteristics of the patients according to the early death are summarized in Supplementary Table 1.

The patients were randomly divided into the training (n = 3070) and validation (n = 1316) cohorts. The demographic and clinical features of the SMPCC patients in the training and validation cohorts are summarized in Table 1. There were no significant differences in demographic and clinical characteristics between the training and validation cohorts.

**Table 1:** Demographic and clinical characteristics of the training and validation cohorts

Variables	Training cohort (n = 3070)	Validation cohort (n = 1316)	p-value
Sex			
Female	1408 (45.86%)	613 (46.58%)	0.686
Male	1662 (54.14%)	703 (53.42%)	
Age(years)			
< 50	226 (7.36%)	113 (8.59%)	0.733
50–59	397 (12.93%)	167 (12.69%)	
60–69	667 (21.73%)	285 (21.66%)	
70–79	891 (29.02%)	390 (29.64%)	
80–89	793 (25.83%)	324 (24.62%)	
≥ 90	96 (3.13%)	37 (2.81%)	
Race			
White	2424 (78.96%)	1054 (80.09%)	0.640
Black	259 (8.44%)	109 (8.28%)	
Other	387 (12.61%)	153 (11.63%)	
Marital			
Married	1595 (51.95%)	677 (51.44%)	0.942
UnMarried	1367 (44.53%)	591 (44.91%)	
Unknown	108 (3.52%)	48 (3.65%)	
Tumor number			
2	2867 (93.42%)	1220 (92.78%)	0.478
≥ 3	202 (6.58%)	95 (7.22%)	
Tumor position			
Unilateral group	1013 (33.02%)	453 (34.42%)	0.385
Bilateral/ rectum-colon synchronous group	2055 (66.98%)	863 (65.58%)	
Histology			
AC	2644 (86.12%)	1118 (84.95%)	0.333
MAC/SRCC	426 (13.88%)	198 (15.05%)	
Grade			
Well/moderately	2223 (72.41%)	934 (70.97%)	0.554
Poorly/undifferentiated	753 (24.53%)	336 (25.53%)	
Unknown	94 (3.06%)	46 (3.50%)	
T stage			
T1	680 (22.15%)	285 (21.66%)	0.563
T3	1836 (59.80%)	799 (60.71%)	
T4	428 (13.94%)	169 (12.84%)	
TX	126 (4.10%)	63 (4.79%)	
N stage			
N0	1659 (54.04%)	687 (52.20%)	0.327
N1	854 (27.82%)	385 (29.26%)	
N2	536 (17.46%)	222 (16.87%)	
NX	21 (0.68%)	22 (1.67%)	
M stage			
M0	2626 (85.54%)	1125 (85.49%)	1.000
M1	444 (14.46%)	191 (14.51%)	
Tumor size(mm)			
≤ 50	1641 (53.45%)	714 (54.26%)	0.533
> 50	1135 (36.97%)	490 (37.23%)	
Unknown	294 (9.58%)	112 (8.51%)	
Chemotherapy			
Yes	1066 (34.72%)	457 (34.73%)	1.000
No/Unknown	2004 (65.28%)	859 (65.27%)	
Radiotherapy			
Yes	334 (10.88%)	118 (8.97%)	0.064
No/Unknown	2736 (89.12%)	1198 (91.03%)	
Surgery			
Non-extensive excision	2707 (88.18%)	1161 (88.22%)	1.000
Extensive resection	363 (11.82%)	155 (11.78%)	

Extensive resection: total colectomy or total proctocolectomy; non-extensive excision: segmental rection, subtotal colectomy, left and right hemicolectomy, total proctectomy

*AC* adenocarcinoma, *MAC* mucinous or mucin-producing adenocarcinoma, *SRCC* signet ring cell carcinoma.

### Risk factors for early all-cause and cancer-specific early death

The univariate logistic regression showed that age, chemotherapy, radiotherapy, histologic type, differentiation grade, T stage, N stage, M stage, and tumor size were associated with early all-cause and cancer-specific early death. Marital status was associated with cancer-specific early death. After performing multivariate logistic regression analysis on the above variables, age chemotherapy, radiotherapy, T stage, N stage, and M stage were identified as independent risk factors for all-cause and cancer-specific early death. In addition, marital status was identified as an independent risk factor for all-cause early death, while the histological grade was identified as an independent risk factor for cancer-specific early death (Tables 2 and 3).

**Table 2:** The univariable and multivariate logistic regression analysis of all-cause early death

Variables	Univariate analysis			Multivariate analysis		
	OR	95% CI	p-value	OR	95% CI	p-value
Sex						
Female	Reference					
Male	0.68	0.53–0.88	0.003	1.01	0.76–1.36	0.92
Age(years)						
< 50	Reference					
50–59	2.12	0.59–7.67	0.253	2.15	0.57–8.03	0.256
60–69	4.24	1.29–13.9	0.017	4.12	1.22–13.9	0.023
70–79	7.43	2.33–23.74	0.001	6.4	1.95–21.06	0.002
80–89	14.98	4.73–47.47	< 0.001	9.67	2.95–31.73	< 0.001
≥ 90	12.69	3.56–45.26	< 0.001	6.11	1.63–22.96	0.007
Race						
White	Reference					
Black	0.95	0.61–1.49	0.832			
Other	0.82	0.55–1.22	0.331			
Marital						
Married	Reference					
UnMarried	1.83	1.42–2.36	< 0.001	1.35	1.01–1.81	0.043
Unknown	0.94	0.43–2.08	0.888	0.75	0.31–1.77	0.505
Tumor number						
2	Reference					
≥ 3	1.53	1–2.36	0.052			
Tumor position						
Unilateral group	Reference					
Bilateral / rectum -colon synchronous group	1.01	0.78–1.32	0.916			
Histology						
AC	Reference					
MAC/ SRCC	1.49	1.08–2.05	0.015	1.25	0.87–1.78	0.224
Grade						
Well/moderately	Reference					
Poorly/undifferentiated	1.56	1.19–2.04	0.001	1.21	0.89–1.64	0.218
Unknown	1.37	0.7–2.68	0.362	1.62	0.75–3.51	0.218
T stage						
T1/2	Reference					
T3	1.52	1.06–2.2	0.024	1.33	0.88–2.01	0.176
T4	2.65	1.73–4.07	< 0.001	2.02	1.21–3.39	0.007
TX	5.28	3.12–8.92	< 0.001	1.73	0.86–3.49	0.124
N stage						
N0	Reference					
N1	1.62	1.21–2.16	0.001	1.81	1.31–2.51	< 0.001
N2	2.1	1.54–2.88	< 0.001	2.44	1.65–3.61	< 0.001
NX	0.68	0.09–5.09	0.705	0.35	0.04–2.91	0.331
M stage						
M0	Reference					
M1	3.17	2.4–4.18	< 0.001	3.61	2.44–5.35	< 0.001
Tumor size(mm)						
≤ 50	Reference					
> 50	1.48	1.14–1.91	0.003	1.26	0.94–1.68	0.123
Unknown	0.95	0.59–1.52	0.824	1.45	0.86–2.46	0.165
Chemotherapy						
Yes	Reference					
No/Unknown	6.02	3.96–9.14	< 0.001	7.11	4.45–11.36	< 0.001
Radiotherapy						
Yes	Reference					
No/Unknown	12.33	3.93–38.69	< 0.001	4.45	1.33–14.87	0.015
Surgery						
Non-extensive excision	Reference					
Extensive resection	1.24	0.87–1.78	0.233			

Extensive resection: total colectomy or total proctocolectomy; non-extensive excision: segmental rection, subtotal colectomy, left and right hemicolectomy, total proctectomy

*AC* adenocarcinoma, *MAC* mucinous or mucin-producing adenocarcinoma, *SRCC* signet ring cell carcinoma.

**Table 3:** The univariable and multivariate logistic regression analysis of cancer-specific early death

Variables	Univariate analysis			Multivariate analysis		
	OR	95% CI	p-value	OR	95% CI	p-value
Sex						
Female	Reference					
Male	0.64	0.48–0.87	0.005	0.99	0.69–1.42	0.963
Age(years)						
< 50	Reference					
50–59	2.88	0.63–13.27	0.174	3.11	0.65–14.94	0.157
60–69	4.4	1.03–18.71	0.045	4.65	1.05–20.58	0.043
70–79	6.39	1.54–26.51	0.011	6.31	1.46–27.26	0.014
80–89	14.19	3.46–58.13	< 0.001	10.69	2.49–45.94	0.001
≥ 90	14.96	3.25–68.92	0.001	8.21	1.65–40.76	0.010
Race						
White	Reference					
Black	1.1	0.66–1.85	0.713			
Other	0.77	0.46–1.27	0.301			
Marital						
Married	Reference					
UnMarried	1.85	1.35–2.52	< 0.001	1.31	0.91–1.89	0.142
Unknown	1.05	0.41–2.65	0.922	0.77	0.27–2.21	0.626
Tumor number						
2	Reference					
≥ 3	1.54	0.92–2.6	0.103			
Tumor position						
Unilateral group	Reference					
Bilateral group/ rectum-colon synchronous group	1.12	0.81–1.55	0.505			
Histology						
AC	Reference					
MAC/ SRCC	1.6	1.09–2.34	0.016	1.28	0.83–1.96	0.265
Grade						
Well/moderately	Reference					
Poorly/undifferentiated	2.15	1.57–2.94	< 0.001	1.6	1.12–2.29	0.010
Unknown	1.66	0.75–3.69	0.21	2.11	0.85–5.23	0.105
T stage						
T1/2	Reference					
T3	1.84	1.11–3.04	0.017	1.3	0.74–2.27	0.362
T4	4.11	2.37–7.14	< 0.001	2.23	1.16–4.29	0.016
TX	8.41	4.44–15.93	< 0.001	1.57	0.69–3.59	0.280
N stage						
N0	Reference					
N1	2.12	1.47–3.06	< 0.001	2.11	1.4–3.17	< 0.001
N2	3.26	2.24–4.75	< 0.001	2.9	1.82–4.63	< 0.001
NX	1.32	0.17–9.99	0.789	0.56	0.06–4.82	0.596
M stage						
M0	Reference					
M1	5	3.64–6.85	< 0.001	5.22	3.38–8.07	< 0.001
Tumor size(mm)						
≤ 50	Reference					
> 50	1.75	1.28–2.39	0.001	1.42	0.99–2.02	0.054
Unknown	0.94	0.51–1.71	0.834	1.47	0.75–2.89	0.264
Chemotherapy						
Yes	Reference					
No/Unknown	4.73	2.95–7.57	< 0.001	6.7	3.93–11.42	< 0.001
Radiotherapy						
Yes	Reference					
No/Unknown	11.86	2.93–48.02	0.001	5.05	1.14–22.34	0.033
Surgery						
Non-extensive excision	Reference					
Extensive resection	1.24	0.8–1.91	0.344			

Extensive resection: total colectomy or total proctocolectomy; non-extensive excision: segmental rection, subtotal colectomy, left and right hemicolectomy, total proctectomy

*AC* adenocarcinoma, *MAC* mucinous or mucin-producing adenocarcinoma, *SRCC* signet ring cell carcinoma

### Construction of the nomogram

**Fig. 2:**
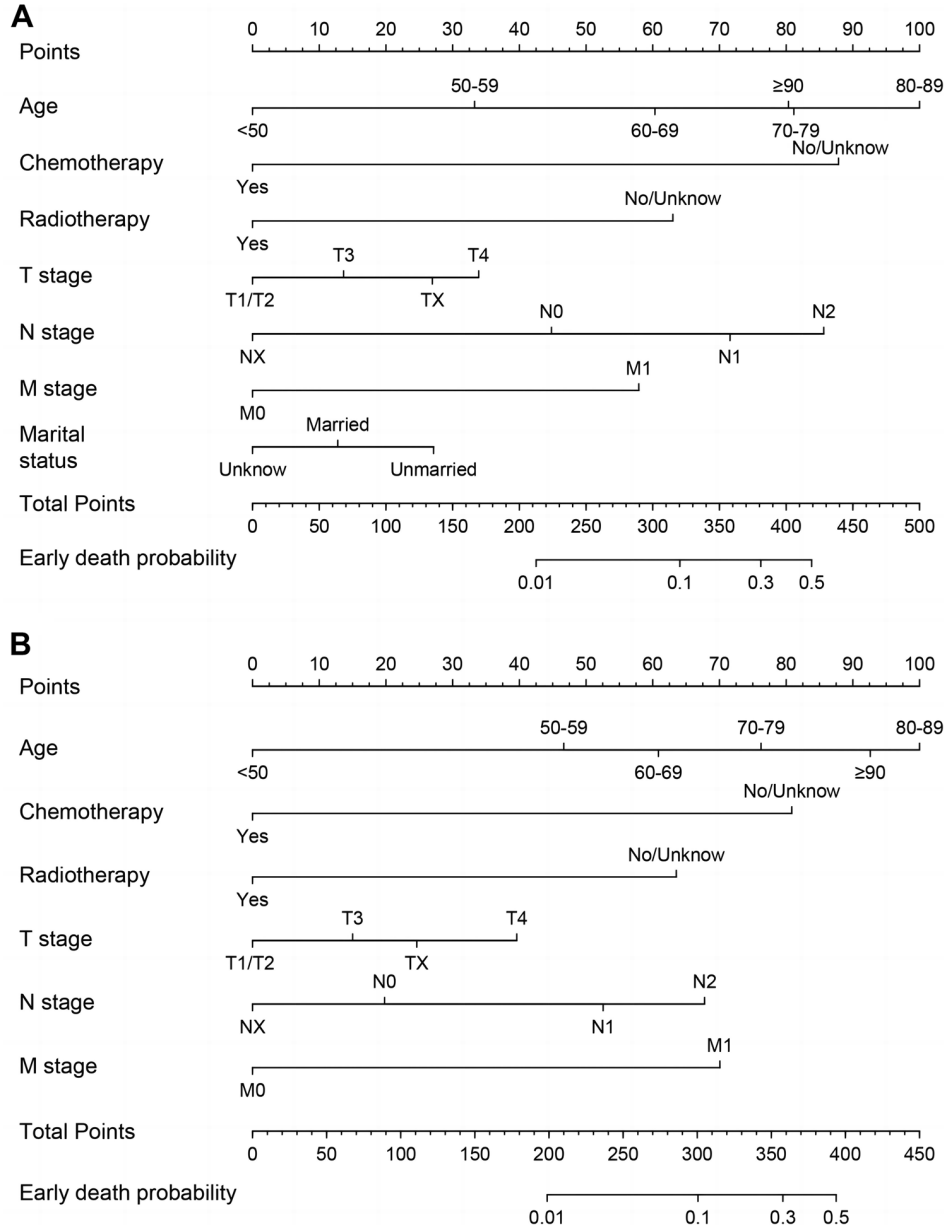
Prediction nomogram of all-cause early death (**A**) and cancer-specific early death (**B**) for SMPCC patients with surgery

The independent risk factors for all-cause and cancer-specific early death were used to construct the predictive nomograms for SPMCC (Fig. 2A, B). The nomograms show the scores corresponding to each risk factor, and the total point represents the sum of all variable scores. The risk for developing all-cause and cancer-specific early death can be found by drawing a line from the total points to the risk score.

### Performance of the nomogram

**Fig. 3:**
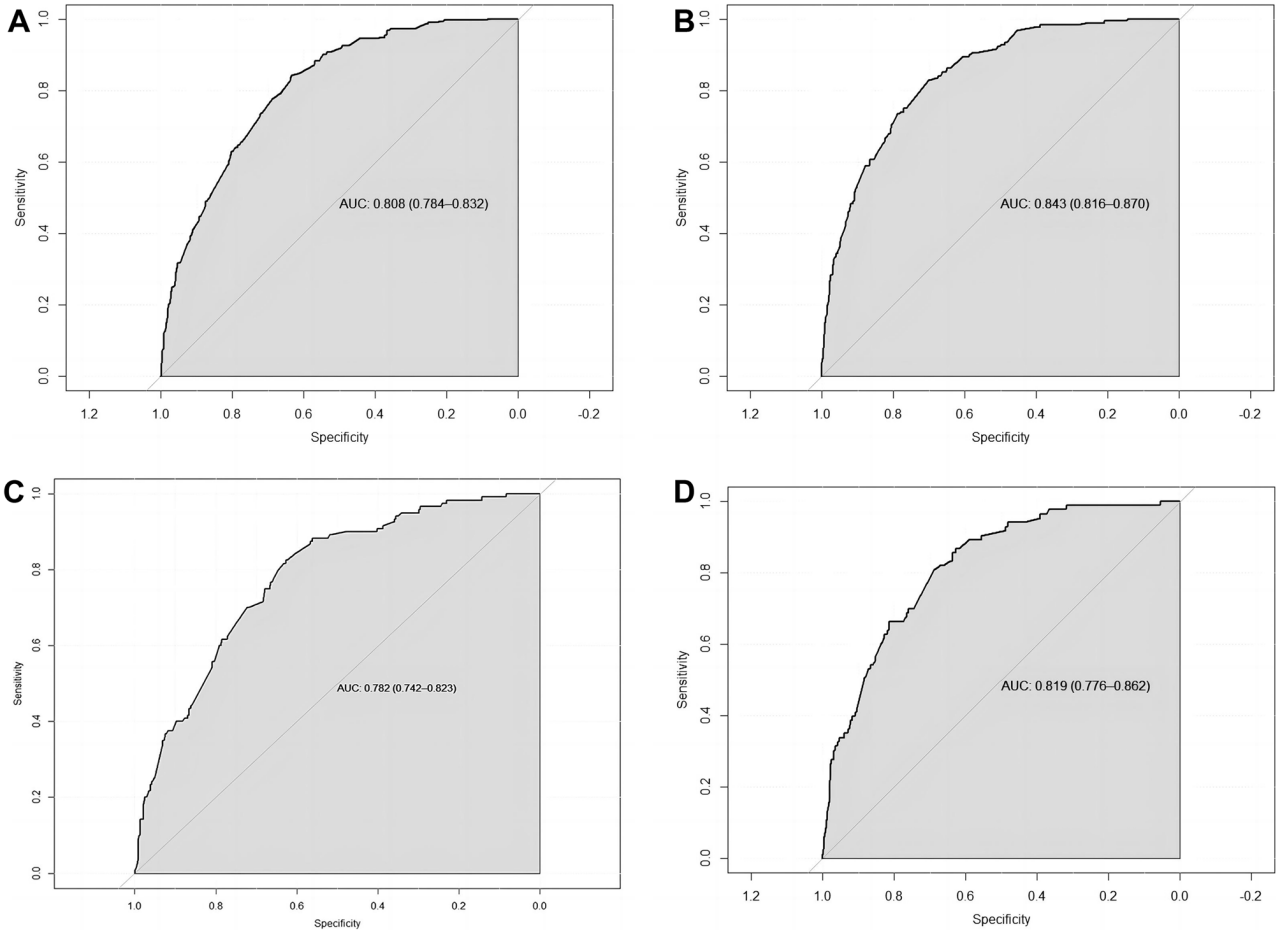
Calibration curves of nomograms for early death. Red line is the performance of nomogram. Blue line corrects for any bias in nomogram. The diagonal line represents a perfect prediction by an ideal model. **A** Calibration curve of all-cause early death in the training cohort. **B** Calibration curve of cancer-specific early death in the training cohort. **C** Calibration curve of all-cause early death in the validation cohort. **D** Calibration curve of cancer-specific early death in the validation cohort

**Fig. 4:**
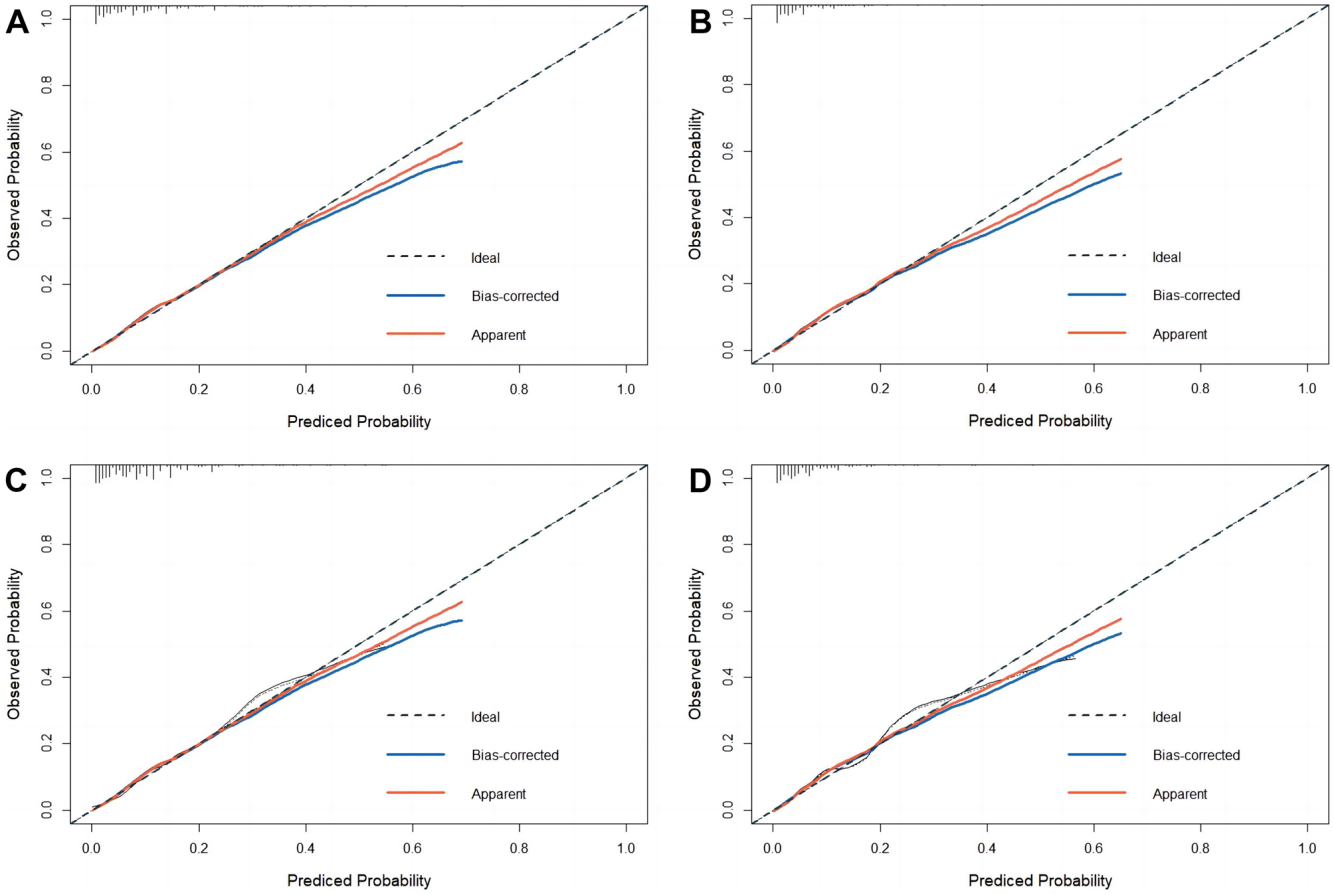
ROC curves of nomogram for predicting early death. **A** ROC curve of all-cause early death in the training cohort. **B** ROC curve of cancer-specific early death in the training cohort. **C** ROC curve of all-cause early death in the validation cohort. **D** ROC curve of cancer-specific early death in the validation cohort

**Fig. 5:**
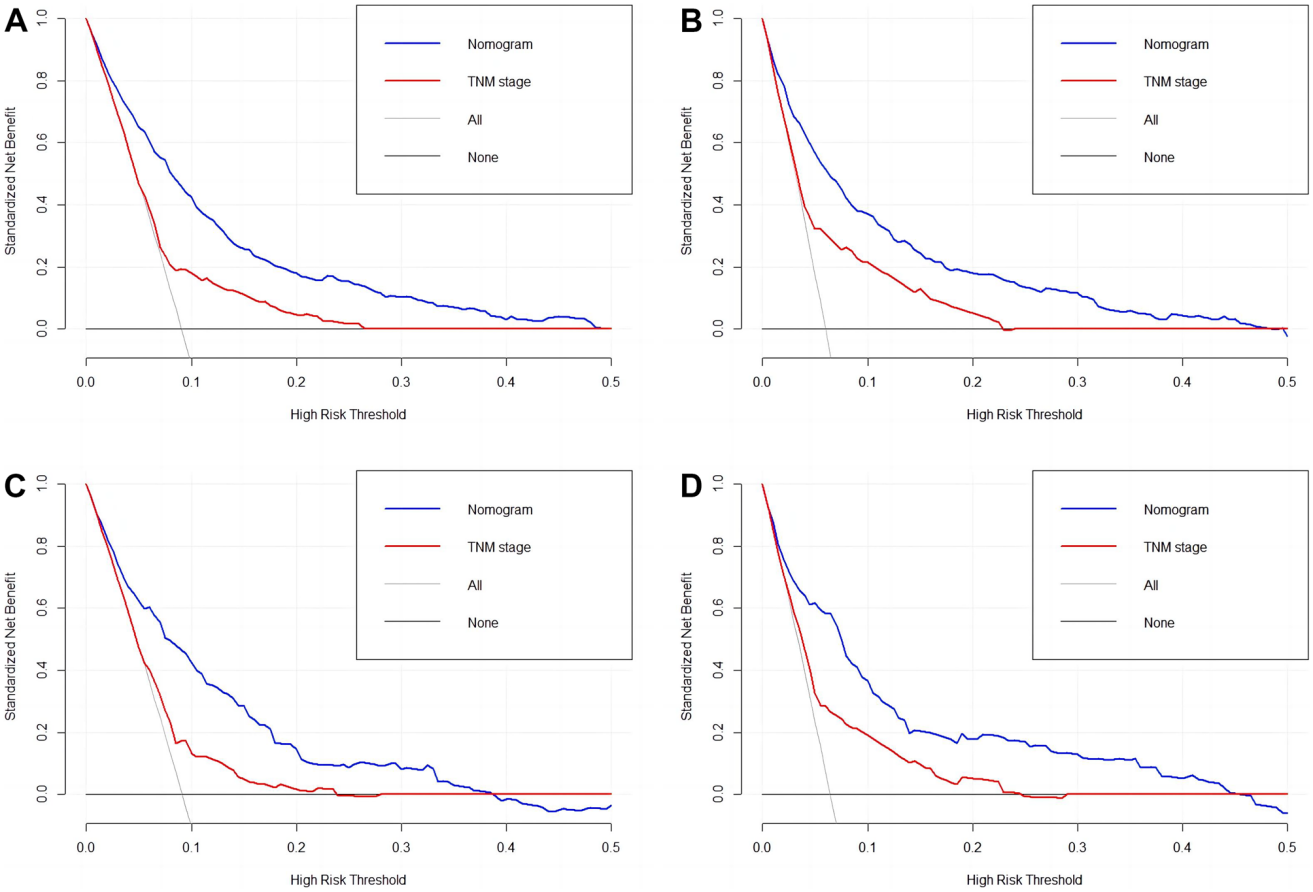
The decision curve analysis (DCA) curves of nomograms for early death, the nomograms (blue line) had a better clinical net value than the TNM staging system (red line). **A** DCA curve of all-cause early death in the training cohort. **B** DCA curve of cancer-specific early death in the training cohort. **C** DCA curve of all-cause early death in the validation cohort. **D** DCA curve of cancer-specific early death in the validation cohort

**In the training cohort, the nomogram achieved a C-index of 0.808 (95% CI, 0.784–0.832) and 0.843 (95% CI, 0.816–0.870) for all-cause and cancer-specific early death, respectively. After validation, the nomogram achieved a C-index of 0.797 (95% CI, 0.758–0.837) and 0.832 (95% CI, 0.789–0.875) for all-cause and cancer-specific early death, respectively.** As shown in the calibration curves, the nomogram achieved considerable agreement between the predicted and actual observations in both training and validation cohorts since the prediction curves are close to the diagonal line (Fig. 3). The AUC values in the training cohort for all-cause and cancer-specific early death were 0.808 (95% CI, 0.784–0.832, Fig. 4A) and 0.843 (95% CI, 0.816–0.870, Fig. 4B), respectively. Following validation, the nomogram achieved an AUC of 0.782 (95% CI, 0.742–0.823, Fig. 4C) and 0.816 (95% CI, 0.779–0.862, Fig. 4D) for all-cause and cancer-specific early death, respectively. The DCA showed that compared to the TNM AJCC staging system, the nomograms achieved a better net benefit for predicting all-cause and cancer-specific early death in both training and validation cohorts (Fig. 5).

## Discussion

SMPCC is a rare CRC subtype characterized by multiple primary synchronous tumors within the colon and rectum. The pathogenesis of SMPCC remains unclear and tends to differ from that of SPCRC. Studies have reported that patients with inflammatory bowel diseases, high microsatellite instability (MSI-H), high CpG island methylation phenotype, hereditary non-polyposis, and familial adenomatous polyposis have a higher risk of developing SMPCC [11, 17, 18]. Surgery is considered the primary treatment option for SMPCC. However, SMPCC patients are more likely to suffer from postoperative complications and early death than SPCRC as they tend to require more extensive surgery [9, 19]. Therefore there is a need to identify survival risk factors for SMPCC to optimize the treatment for these patients. To the best of our knowledge, this is the first study to construct a prognostic prediction nomogram that could be used to predict all-cause and cancer-specific early death in patients with resected SMPCC.

In this study, we extracted the clinical data of 4386 SMPCC patients from the SEER database. Of these patients, 9.07% and 6.00% died due to all-cause and cancer-specific cause. The univariate and multivariate logistic regression analysis identified older age, no or unknown chemotherapy, no or unknown radiotherapy, and higher TNM stage as predictive risk factors for all-cause and cancer-specific early death. While the risk of early death from all-cause was higher in unmarried patients, the risk of early death from cancer-specific cause was higher for patients diagnosed with poorly or undifferentiated grade.

Our predictive nomograms based on the above risk factors achieved good predictive performance for early death in both training and validation cohorts. In addition, our nomogram achieved a higher clinical net benefit than the standard AJCC TNM staging system, thus confirming the clinical value of the nomogram. The AJCC TNM staging system is widely used to predict the prognosis of CRC. Previous studies have shown that tumor invasion depth, lymph node metastasis, and distant organ metastasis are associated with early death in patients with CRC undergoing surgery [20]. Consistent with these studies, our research has identified these three factors as predictive of early death in SMPCC patients. Consistent with previous studies, 79.14% of SMPCC patients in our study were aged 60 years or more and had a higher mean age than SPCC patients [21]. Similarly to our findings, previous studies have shown that advanced age is a risk factor for poor short-term and long-term prognosis in CRC patients [22, 23]. Older adults are more likely to present with comorbidities, poor physical status, and a higher incidence of preoperative intestinal obstruction and perforation than younger patients. Moreover, older adults are also more likely to require emergency surgery [24, 25]. As a result, elderly patients are more at risk of developing postoperative complications and mortality than younger patients [26, 27]. In addition, older patients are less likely to tolerate radiotherapy and chemotherapy due to their poor physical status. Chronic diseases such as heart failure, diabetes, and chronic obstructive pulmonary disease are more common in elderly patients [28, 29]. Therefore, elderly patients have an increased risk of dying from non-cancer-specific causes. Therefore, the treatment of elderly SMPCC patients undergoing surgery needs to be optimized to reduce the risk of early death.

There is still a lack of consensus on the optimal postoperative adjuvant therapy for SMPCC patients, particularly for stage II disease. Some studies suggest that SMPCC patients are more at risk of developing micrometastases than SPCRC patients and hence are more likely to benefit from postoperative adjuvant therapy [30]. On the other hand, some studies argue that since SMPCC patients are more likely to exhibit MSI-H (high microsatellite instability) or dMMR (deficient MMR) than SPCRC patients, they are less likely to benefit from fluorouracil-based chemotherapy [31]. Our study showed that both chemotherapy and radiotherapy reduced the risk of early death in SMPCC patients. For SMPCC patients undergoing surgery at risk of early death, adjuvant therapy such as radiotherapy and chemotherapy should be considered depending on the individual circumstance. However, larger clinical randomized controlled studies are required to identify the optimal adjuvant therapy for SMPCC patients.

Similar to previous studies, married patients were less at risk of developing early death [32]. Married patients are more likely to receive physical and financial support from their partners to cope with the disease, and, therefore, they are less likely to suffer from early death. Moreover, consistent with previous studies, we also found that poorly differentiated or undifferentiated tumors are more at risk of developing early mortality due to a higher risk of metastasis [33].

Our findings suggested that extensive resection (total colectomy or proctocolectomy) did not increase the risk of early death in SMPCC patients. Currently, there is no consensus on the extent of surgical resection for SMPCC patients. Hemicolectomy or extended hemicolectomy should be considered for tumors located in the same or adjacent segment. However, extensive resection (total colectomy or proctocolectomy) or multiple segmental resections with synchronous bowel anastomoses are recommended if the tumors were localized in distant segments [19]. Some studies have demonstrated that extensive resection can improve prognosis in SMPCC patients compared with multiple.segmental resections. However, due to the small sample size in this study, the conclusions need to be further verified [34, 35]. In addition, extensive resection for SMPCC patients with high-risk factors, including; familial adenomatous polyposis, inflammatory bowel disease, or hereditary non-adenomatous colorectal cancer was recommended by most studies [36, 37].

This study has several limitations that have to be acknowledged. The data were retrospectively extracted from the SEER database. The lack of quality control in the data included in the SEER database may have biased our results. In addition, we could not explore the association of other potential risk factors for early death in SMPCC, such as nutritional status, carcinoembryonic antigen (CEA), and susceptibility factors (inflammatory bowel disease, familial adenomatous polyposis, and hereditary non-adenomatous colorectal cancer) as this information was not reported in the SEER database. Finally, since the data was collected from a single database, further research is required to validate the generalizability of the nomogram in multiple centers.

## Conclusion

In this study, we developed a novel risk prediction nomogram for early all-cause and CS survival in patients with resected SMPCC using data extracted from the SEER database. The nomogram achieved high prediction accuracy and consistency in both training and validation cohorts. The DCA showed that the nomograms had a better clinical net value than the TNM staging system. This model can provide a simple and accurate tool for clinicians to predict the risk of early death in SMPCC patients undergoing surgery and could be used to optimize the treatment according to the patient's needs.

### Supplementary Information

Below is the link to the electronic supplementary material.

Supplementary file1 (XLS 32 KB)

## Data Availability

The data that support the findings of this study are available from the Surveillance, Epidemiology, and End Results (SEER) database at http://www.seer.cancer.gov.
